# Reliability, validity, and clinical utility of a culturally modified Kessler scale (MK-K5) in the Aboriginal and Torres Strait Islander population

**DOI:** 10.1186/s12889-021-11138-4

**Published:** 2021-06-10

**Authors:** Makayla-May Brinckley, Bianca Calabria, Jennie Walker, Katherine A. Thurber, Raymond Lovett

**Affiliations:** National Centre for Epidemiology & Population Health, Research School of Population Health, Australian National University, 54 Mills Road, Acton, ACT 2601 Australia

**Keywords:** Aboriginal, Torres Strait Islander, Kessler, Psychological distress, Mixed-methods, Reliability, Validity, ROC curves

## Abstract

**Background:**

Aboriginal and Torres Strait Islander peoples are the first people of Australia. Consequences of historic and contemporary settler-colonialism including racism, trauma, grief and loss (of land, culture, spirituality, and freedoms) have led to substantial negative health and wellbeing impacts. The Kessler Psychological Distress Scales are population and individual-level tools designed to measure general psychological health status. There has been limited assessment of the psychometric properties and validity of the Kessler Psychological Distress Scale for use with the Aboriginal and Torres Strait Islander population in Australia, despite its widespread use.

**Methods:**

A national sample of Aboriginal and Torres Strait Islander adults (*n* = 6988 ≥ 16 years) was used in the psychometric assessment of the MK-K5, which involved face validity, acceptability, internal consistency/reliability, construct validity, and convergent and divergent validity testing. Receiver Operator Characteristics (ROC) curves were produced to assess clinical utility for depression and anxiety screening.

**Results:**

The MK-K5 demonstrated face validity for psychological distress in two focus groups, and had good acceptability, good internal consistency/reliability (α = 0.89), good construct validity (uni-dimensional; one underlying component explaining 70.1% of variance), and demonstrated convergent and divergent validity in the sample. The MK-K5 had good clinical utility at a cut-off score of 11 for detecting ever being diagnosed with depression or anxiety.

**Conclusions:**

The MK-K5 is a valid measure of psychological distress and has clinical utility in the Aboriginal and Torres Strait Islander population.

**Supplementary Information:**

The online version contains supplementary material available at 10.1186/s12889-021-11138-4.

## Background

Aboriginal and Torres Strait Islander peoples are the first people of Australia, having lived on and from the land for tens of thousands of years, with archaeological records placing Aboriginal and Torres Strait Islander peoples in Australia at least 65,000 years ago [1]. Colonisation in Australia began in 1788 when the British deemed the country uninhabited and claimed Australia for the British empire, despite Aboriginal and Torres Strait Islander peoples’ existing agriculture, industry, and communities [2–4]. The violent dispossession of Aboriginal and Torres Strait Islander peoples occurred across the continent, including in the most remote inland and island places [3].

Decades of attempted destruction of Aboriginal and Torres Strait Islander families and communities, coupled with contemporary racism, trauma, and marginalisation (including in the healthcare system), have had detrimental impacts on the lives, health, and wellbeing of Aboriginal and Torres Strait Islander peoples [5, 6]. One stark example is the Stolen Generation; Aboriginal and Torres Strait Islander children were forcibly removed from their families under Government policies and forced to assimilate into non-Indigenous Australia, which included being forbidden to speak their languages or practice culture [7]. People removed during the Stolen Generation and their descendants today experience poorer health and wellbeing outcomes, lower educational and socioeconomic outcomes, and increased trauma, than those not removed [8–10].

Historic racism and trauma and their ongoing negative effects on Aboriginal and Torres Strait Islander people is further demonstrated by the disproportionate burden of mortality and morbidity experienced by the population [11]. In 2014–15, 65.0% of Aboriginal and Torres Strait Islander participants had a long-term health condition, including 29.3% of participants who reported a diagnosed mental health condition [12]. Overall, 38.4% of participants had high to very high levels of psychological distress, as measured by the K5 [12]. High to very high levels of psychological distress were around three times as common among those reporting a diagnosed mental health condition (59.6%) compared to those not reporting a mental health condition (21.0%) [12].

Psychological distress can be defined as “a state of emotional suffering associated with stressors and demands that are difficult to cope with in daily life” [13]. A common screening tool for psychological distress internationally is the Kessler Psychological Distress Scale, a short-form psychometric scale. The Kessler Psychological Distress Scale was developed in 1992 using a United States population sample [14]. The diverse cognitive, behavioural, emotional and psychophysiological processes evident in a variety of mental illnesses were found to load onto one single factor, referred to as non-specific psychological distress. As this non-specific psychological distress factor is present in a wide range of disorders, and population level measurement of psychological distress was needed, the Kessler Psychological Distress Scale was developed as a unidimensional measure of population level non-specific psychological distress [15].

There are three scales: the Kessler 10 (K10; with 10 items), the shorter version Kessler 6 (K6; with 6 items) [14], and the 5-item Kessler Psychological Distress Scale (K5) which was developed in consultation with Aboriginal and Torres Strait Islander stakeholders at a 2003 social and emotional wellbeing workshop as a culturally sensitive subset of the K10 for use in the National Aboriginal and Torres Strait Islander Health Survey (NATSIHS). This was in response to concerns that asking someone the amount of time they felt “worthless” would be culturally inappropriate and offensive to some Aboriginal and Torres Strait Islander people [16, 17]. This adaptation and the resulting K5 was endorsed by Professor Kessler [16]. The adaption of the original K5, however, occurred with members attending a workshop. There is limited information available about the process involved in the adaptation. In this process, the K5 did not receive full psychometric or face validity assessment across a diversity of Aboriginal and Torres Strait Islander peoples [16]. The K5 is used for population-level research purposes as an assessment of Aboriginal and Torres Strait Islander psychological distress, mental health, and wellbeing. See Table 1 for a comparison of the Kessler Psychological Distress Scale versions.

**Table 1:** Comparison of items in the K10, K6 and K5

K10	K6	K5
*In the past 4 weeks how often have you felt…*	*In the past 4 weeks how often have you felt…*	*In the last 4 weeks how often have you felt…*
1 Tired out for no good reason		
2 Nervous	1 Nervous	1 Nervous
3 So nervous nothing could calm you down		
4 Hopeless	2 Hopeless	2 Without hope
5 Restless or fidgety	3 Restless or fidgety	3 Restless or jumpy
6 So restless you could not sit still		
7 Depressed	4 So depressed that nothing could cheer you up	
8 That everything was an effort	5 That everything was an effort	4 That everything was an effort
9 So sad that nothing could cheer you up		5 So sad that nothing could cheer you up
10 Worthless	6 Worthless	

Previous research has assessed the validity of the Kessler Psychological Distress Scales in various populations, including the general Australian population [18]. However, there is limited research on the validity of the Kessler Psychological Distress Scales in the Aboriginal and Torres Strait Islander population [19, 20].

While some exploratory validation work has been completed, a full psychometric evaluation of the culturally-modified K5 has not yet occurred [16]. The validity of the K10, and the original (not culturally modified) K10 items comprising the K5, was investigated among 1631 Aboriginal and/or Torres Strait Islander adults and 231,774 non-Indigenous adults aged 45 years and older who completed the baseline survey of the 45 and Up Study in New South Wales, Australia [21]. The results found agreement in the classification of distress between the K10 and unmodified K5 items (*p* = 0.54) among Aboriginal and Torres Strait Islander adults. It also found that the unmodified K5 had adequate internal consistency (Cronbach’s α = 0.88) and the K10 had some item redundancy (Cronbach’s α = 0.93). A confirmatory factor analysis supported the theory of a single underlying factor structure for both measures [21]. This study demonstrated internal and construct validity of the K10 and unmodified K5 for measuring unidimensional psychological distress among Aboriginal and Torres Strait Islander people aged 45 years and older.

While this study found the K10 and the unmodified K5 items to be valid in this sample, there are a number of potential limitations to the study. First, the survey is not focused on the Aboriginal and Torres Strait Islander population and therefore they did not use the K5 in its culturally adapted form, but used the original wording from the K10. Second, the validation study only involved Aboriginal and/or Torres Strait Islander people aged 45 years and over residing in NSW. Further, while the study suggests that the unmodified K10 items that comprise the K5 is valid in this Aboriginal and Torres Strait Islander sample, it does not assess the face validity nor does it assess the cultural appropriateness of the items. As such, there is a need for the K5 to undergo face validity testing to assess the cultural applicability of the measure alongside this statistical validity testing. Therefore, we currently lack robust evidence of validity for the culturally adapted K5 for the Aboriginal and Torres Strait Islander population, in particular for younger adults and those living in other contexts.

Validity of the Kessler Psychological Distress Scales has critical implications for clinical use, due to its widespread use with Aboriginal and Torres Strait Islander people in primary care to indicate referral to further clinical treatment [22]. Using measures in the healthcare system without knowing if they are valid and reliable poses risks, including patients missing referral for crucial treatment. For an effective healthcare system, psychometric health and wellbeing measurement tools, including the K5, must be validated for the population they are used with. This supports appropriate health screening and referral for further assessment where indicated.

To contribute to the literature on the validity and clinical utility of the K5, we assessed the validity of an adapted K5 (MK-K5) in a national sample of Aboriginal and Torres Strait Islander adults aged ≥16 years. We examined face validity, acceptability, internal consistency/reliability, construct validity, and convergent and divergent validity. To assess the clinical utility of the MK-K5, we utilised ROC curves to determine cut-off scores in detecting depression and anxiety.

## Methods

### Sample

Mayi Kuwayu: the National Study of Aboriginal and Torres Strait Islander Wellbeing (Mayi Kuwayu Study) is a longitudinal study of culture and wellbeing [23]. Participants of the current study are Aboriginal and/or Torres Strait Islander adults aged ≥16 years, recruited via postal questionnaire (identified through the Medicare Enrolment Database), in-community recruitment, community partners, or online questionnaire, from October 2018 to August 2019 (*n* = 7526, Data Release 1.0). This analysis was restricted to the 6988 participants with a total MK-K5 score; 538 participants missing a response on one or more items were excluded. All data analysed in this study are based on self-reported responses to the baseline questionnaire. All participants gave their consent for participation and publication. Young people aged 16–17 years old are additionally covered by ethics committee approvals with justification in line with sections 4.2.8 and 4.2.9 of the National Statement on Ethical Conduct in Human Research [24]. All methods were carried out in accordance with guidelines and regulations from the Australian Institute of Aboriginal and Torres Strait Islander Studies (AIATSIS).

### Measures

The Mayi Kuwayu Study undertook extensive face validity assessment as part of survey development, including the K5 [23, 25]. This involved hosting focus groups at participating community organisations (for example Aboriginal health services) across saltwater, freshwater, desert and Island groups, in urban, regional, and remote areas of Australia, representing the high degree of diversity and cultural experiences across Aboriginal and Torres Strait Islander peoples. This process involved 28 focus groups with 197 participants who pre-tested the survey questions (Supplementary Table S2).

The K5 was adapted in response to feedback received during the initial face validity process, so the K5 used in the Mayi Kuwayu Survey (MK-K5) includes slight wording changes and clarifying statements, therefore varying from the original K5 (Table 2). “So sad that nothing could cheer you up” was reduced to “Sad” in the MK-K5 due to a lack of understanding of the phrase “nothing could cheer you up” in the original K5 item. The clarifying statements were added to increase conceptual understanding of the items. However, since the concepts in each item remain the same and the clarifying statements’ purpose is to increase understanding by participants, findings from this measure can still be compared to other literature on the K5.

**Table 2:** Comparison of the K5 items and the Mayi Kuwayu Study adapted K5 items (MK-K5)

K5	MK-K5
Nervous	Nervous
Without hope	Hopeless (have no hope)
Restless or jumpy	Restless or jumpy
That everything was an effort	Everything was an effort (have no energy)
So sad that nothing could cheer you up	Sad

K5 scores range from 5 to 25, with higher scores indicating higher level of psychological distress [17]. The categories and their cut-off scores used in the NATSIHS are 5–< 8 for low, 8–< 12 for moderate, 12–< 15 for high, and 15–25 for very high psychological distress [16]. These categories have been applied to this analysis to describe population level psychological distress. To our knowledge, these cut-offs were not based on risk for adverse mental health outcomes, and are instead used to define categories for use in population-level research.

Variables used for assessment of convergent and divergent validity were self-reported lifetime doctor diagnosis of depression and anxiety, happiness over the past 4 weeks (categorised as low or high happiness), and self-reported lifetime doctor diagnosis of heart disease (see Table S1 for details).

### Analyses

#### Sample characteristics

Demographic characteristics of the sample were explored, overall and in relation to K5 category, with Pearson’s chi-squared test used to test if variables were independent.

#### Face validity

Face validity was assessed to determine whether participants were interpreting the items in the K5 as intended [26]. In addition to the initial face validity assessment during the survey development process [25], this study conducted two focus groups in August 2019 (total *n* = 9). All focus group participants were Aboriginal and/or Torres Strait Islander university staff and students aged ≥16 years who were purposefully recruited [27]. Email invitations were sent through two university Indigenous email groups indicating the focus group dates. Participation was voluntary and following an overview of the Mayi Kuwayu Study, each participant provided written informed consent to participate.

Focus groups were held in private rooms in locations known to be culturally safe on the university campus [28]. The researcher conducting the focus groups was an Aboriginal researcher with experience in conducting focus groups. Participants were asked how they would describe each word from the items in the MK-K5. Steps taken to guard against researcher selectivity and bias included consistency in the wording of questions asked and remaining neutral to participants’ responses.

The total time for each focus group was approximately 60 min. Focus groups were audio recorded and transcribed by Type Transcripts [29]. The qualitative analysis of focus group data aimed to describe each MK-K5 concept and draw any major links about the concepts more generally from each focus group, using a framework analysis and a thematic approach, as outlined by Rabiee [27], supported by NVivo Version 11 [30].

### Statistical analyses

An alpha level of 0.05 for all statistical tests was considered significant.

#### Acceptability

Acceptability was assessed through examination of missing data across each item and the entire scale. Missing data of <10% was considered desirable [31].

#### Internal consistency/reliability

Cronbach’s alpha was used to assess internal consistency, measuring homogeneity of items [32].

#### Construct validity

For construct validity, Principal Components Analysis (PCA) was used to determine the number of underlying components the measurement items are related to [33]. Exploratory Factor Analysis has previously been done in this population to establish the factor structure of the unmodified K5 [34], so in this paper we utilise the PCA as a confirmatory approach to construct validity.

#### Convergent and divergent validity

Convergent validity was assessed using outcomes understood to be conceptually related to psychological distress or its absence (Depression, Anxiety, Happiness). It was expected that Depression and Anxiety would be positively associated with increasing psychological distress, while Happiness would be negatively associated with psychological distress, as psychological distress and happiness are understood to be at disparate ends of the psychological wellbeing continuum [35].

Psychological distress is conceptually potentially related to most variables available in the Mayi Kuwayu Study dataset. For an assessment of divergent validity, we examined the association between psychological distress and a measure we considered to be less strongly related to psychological distress than in the convergent validity assessment (Heart Disease). It was expected that any association between psychological distress and heart disease would be of weaker magnitude than associations observed with the measures used for convergent validity assessment.

Unadjusted binomial regression was used to assess convergent and divergent validity. Prevalence Ratios (PR) and 95% Confidence Intervals (CIs) for each outcome were quantified, as outcomes were common.

#### Clinical utility

Clinical utility was investigated though Receiver Operator Characteristic (ROC) curve analysis. Self-reported lifetime doctor diagnosis of anxiety or depression was used due to a lack of a gold standard measure (i.e., a current diagnosis reported by a healthcare professional or clinical interview administered by a trained professional) in the dataset.

Sensitivity, specificity, positive-predictive value, and negative-predictive value were calculated for each score on the MK-K5 and data identifying self-reported doctor diagnosed depression and anxiety. General guidelines recommend selecting a cut-off point where sensitivity and specificity are maximised and as close together as possible [36, 37]. In this study, we follow this approach to give equal weight to sensitivity and specificity. This approach was chosen as it balances the risks of not identifying someone who has depression or anxiety, compared to the risks of falsely identifying someone without depression or anxiety, while acknoweldging the strained healthcare system in Australia [38].

## Results

### Participant characteristics

Participants were 6988 Aboriginal and Torres Strait Islander people aged ≥16 years (male = 39.8%, female = 61.0%, other genders = 0.1%). Geographically, 45.8% of participants lived in major cities, 46.7% lived regionally, and 7.4% of participants lived remotely (missing = 1.0%). Forty-seven percent of participants had completed tertiary education as their highest level of education. A total of 3771 participants (54.0%) were employed in paid work, while 722 were unemployed (10.3%) and 1185 participants (17.0%) were retired (Table 3).

**Table 3:** Demographic characteristics of study participants (*N* = 6988)

Variable	n	%
**Age group**		
16–24	601	9.4
25–49	2086	32.7
50+	3687	57.8
**Gender**		
Male	2671	38.9
Female	4193	61.0
Other genders	8	0.1
**Remoteness**		
Major city	3160	45.8
Regional	3221	46.7
Remote	512	7.4
**Education level**		
No school	42	0.6
Primary school	224	3.2
Some high school	1103	15.9
Year 10	1529	22.1
Year 12	796	11.5
Certificate/diploma	1908	27.6
University	1323	19.1
**Employment**^**a**^		
Paid employment	3771	54.0
Unemployed	722	10.3
Retired	1185	17.0
Carer	374	5.6
Studying	726	10.4
CDP	76	1.1
Disabled/sick	810	11.6

^a^Participants could select more than one type of employment and therefore percentages sum to more than 100%

60.9% of participants had low-to-moderate levels of psychological distress, while 39.0% of participants experienced high-to-very high levels of psychological distress, according to NATSIHS cut-offs (Table 4).

**Table 4:** Level of psychological distress, according to MK-K5, overall and in relation to age group, gender, and remoteness

	Low (5- < 8)	Moderate (8- < 12)	High (12- < 15)	Very High (15–25)
	% (n)	% (n)	% (n)	% (n)
**Overall**	29.5 (2063)	31.4 (2195)	15.4 (1079)	23.6 (1651)
**Age Group**				
16–24	19.3 (116)	30.0 (180)	21.1 (127)	29.6 (178)
25–49	25.1 (524)	32.5 (678)	16.4 (341)	26.0 (543)
> 50	34.1 (1256)	31.1 (1145)	14.0 (517)	20.9 (769)
**Gender**^**a**^				
Male	34.5 (921)	30.8 (822)	13.8 (368)	21.0 (560)
Female	26.3 (1104)	31.8 (1335)	16.5 (691)	25.4 (1063)
**Remoteness**				
Major city	28.0 (844)	31.7 (1000)	15.9 (501)	24.5 (775)
Regional	30.0 (965)	30.9 (995)	15.5 (498)	23.7 (763)
Remote	36.5 (187)	33.8 (173)	13.7 (70)	16.0 (82)

^a^Due to small numbers, people identifying as other genders are included in totals but are omitted from gender-stratified analysis

There were significant differences in psychological distress categories by age (*p* < 0.001). The prevalence of psychological distress was highest in the 16–24 age group, with 50.7% of participants having high to very high levels of psychological distress. There were significant differences in psychological distress categories by gender (*p* < 0.001), with 41.9% of women having high to very high levels, compared to 34.8% of men. There were significant differences in psychological distress categories by remoteness (*p* < 0.001). Thirty percent of people living in remote areas have high to very high levels of psychological distress, compared to 39.2% of people in regional Australia, and 40.4% of people in major cities.

### Face validity

#### Nervous

Focus group participants described “nervous” as feeling anxious or scared. They described the common physiological sensations of being nervous. As one participant stated:

Female 1, 16–19 years: *I think nervous is more like a bodily response, like your heart is beating faster.*

Other physical sensations included “butterflies in your stomach” and experiencing “sweaty palms and kind of shallow breathing.”

Finally, participants stated that feeling nervous often comes with the anticipation of events:

Male 1, 20–29 years: *I associate being nervous with having to do something, like having to give a speech or something like that.*

#### Hopeless

Participants described “hopeless” as feeling depressed, defeated, and empty. They described hopelessness “like it’s the end” where people think there’s “no way out”:

Female 2, 60–69 years: *There’s somebody that’s hit rock bottom.*

Female 1, 16–19 years: *Like sit around and think about it.*

Female 3, 20–29 years: *You kind of resign yourself to the fact that you can’t do anything to change it.*

One participant stated that people feeling hopeless are defeated and plan for the end:

Female 4, 16–19 years: *I would imagine you get quite self-destructive, and coming back to isolated, but kind of giving your belongings or your thoughts away, kind of doing the last type things.*

#### Restless

“Restlessness” was described as both a physical and mental experience. Participants described it as feeling physically “fidgety” and constantly moving:

Male 2, 40–49 years: *It’s also repetitive, isn’t it? Like, as soon as you think about someone that’s really restless, the foot, it’s a repetitive movement. It is the same movement or swinging your chair.*

These physical sensations were often described as being “unconscious”. The mental component was described as:

Female 1, 16–19 years: *Over-thinking about something, and I [feel] like I can’t rest.*

Male 1, 20–29 years: *It’s like you can’t really focus; you jump from thing to thing without finishing each of them.*

#### Jumpy

Participants described “feeling jumpy” as a physiological reaction to external events, including the reaction to events:

Male 2, 40–49 years: *So jumpy sort of implies to me that you’re a little bit on edge … I always think of it as a reaction to something.*

There were some discrepancies in the cognitive aspect of experiencing jumpy; where some participants stated that you are aware of your surroundings while others said the opposite:

Female 5, 30–39 years: *You’re almost, like, hyper vigilant. You’re very aware of your surroundings.*

Female 3, 20–29 years: *Like you’re too far in your own head that you don’t notice things going on around you.*

#### Effort

“Feeling as though everything is an effort” was commonly associated with a person who has “shut down” and physically cannot do anything:

Male 2, 40–49 years: *A classic image that comes to mind is you just lay down doing nothing.*

Participants also described how this feeling relates to even the simplest, day-to-day tasks:

Female 4, 16–19 years: *Like a bit crazy, you just feel like previously so busy, and then this next thing, it feels like it takes so much effort that you feel crazy: while washing up the dishes, “why am I going insane washing up the dishes?”*

Finally, they drew links between a normal level of exhaustion and feeling as though everything was an effort:

Female 6, 50–59 years: *It’s not the tired of being for a long walk, “I’ve done a lot of physical activity today” tired - it’s just a bone exhaustion tired.*

#### No energy

Participants described “having no energy” as both a physiological and a cognitive experience:

Female 2, 60–69 years: *So, there can be no physical energy or no mental energy.*

Female 4, 16–19 years: *I was thinking just if you’re physically ill, you feel like you have no energy. But I suppose that goes with the same thing as if you’re mentally unwell as well.*

For physically having no energy, participants stated that it may be due to feeling “overworked” and is similar to feeling as though everything is an effort. When describing mentally having no energy, participants also stated that while people can be mentally tired, it is hard to switch-off:

Female 3, 20–29 years: *I think they can also think about the people that they might be letting down by not doing what they’re supposed to be doing.*

#### Sad

Participants described sadness as a feeling of isolation and aloneness. They discussed the beginning of sadness as “trembling”, and your eyes start “welling up” before you cry. Sadness was described on a continuum of emotions:

Male 1, 20–29 years: *So being sad is something that is further down on the spectrum.*

Sadness was linked to mental disorders:

Female 4, 16–19 years: *I think sadness is linked with mental health, right, because it’s an emotion, but then it’s also like depression and anxiety and that kind of thing because they are like illnesses that kind of exaggerate the sadness.*

Participants spoke about the stigma of feeling sad, especially when related to a mental health issue that prolongs sadness:

Male 1, 20–29 years: *People are hesitant to identify with sadness … people don’t want to say they’ve been sad for such an elongated time.*

#### Overarching themes

Alongside the descriptions of individual MK-K5 concepts, two major themes organically arose from the focus groups: firstly, that the MK-K5 concepts are linked on a continuum and may relate to one underlying concept, and secondly that the MK-K5 concepts are not always negative emotions.

### Links between MK-K5 concepts

Participants drew links between the MK-K5 concepts, often stating how one concept would occur first, with another concept as the “after-effect”:

Male 2, 40–49 years: *What contributes to jumpy and restless could be nervousness or the worry. It could be either of those. So they’re probably related to those two.*

Female 2, 60–69 years: *[Feeling jumpy] does tie in with nervous a lot, a heck of a lot. It’s like an after-effect of nervous, that you’re also jumpy.*

Both focus groups described the MK-K5 items as on a continuum where one leads to another. One example of the concepts being interconnected in a sequential way was described by participants as:

Male 1, 20–29 years: *I think all of them are connected, but especially with this one, when you’ve got no energy, you could still be worried about the all things you’re not doing; you’re nervous about how people are going to react to that; you’re kind of losing hope because you just can’t be fucked to deal with it. Yeah, I think they are all connected in that way.*

Female 6, 50–59 years: *[People] can still function when they’re nervous but by the time they reach a state where they feel hopeless, they’ve pretty well reached a state of “I’m dysfunctional now”.*

### MK-K5 concepts are not always negative

Participants gave many examples of how each concept is not necessarily related to negative emotions:

Female 3, 20–29 years: Or *you get nervous because you’re excited about something. So it can be like a positive sometimes.*

Female 4, 16–19 years: *Yeah, like a new experience.*

Participants also described other factors that may cause these emotions that are not related to psychological distress. For example, participants described experiencing restlessness because of external influences:

Male 2, 40–49 years: *Yeah, and if you physically – if you run a marathon, you’ve definitely got no energy.*

Female 6, 50–59 years: *So, yeah, restlessness can – it’s not necessarily a bad thing … If you’re waiting for somebody or something like that you can feel a little bit restless. It’s not necessarily a negative thing.*

#### Acceptability

Less than 5% of participants were missing individual MK-K5 items; 7.2% were missing the total score (Table 5).

**Table 5:** Missing data for individual MK-K5 items and total score

MK-K5 items	n	%
Nervous	350	4.7
Hopeless (have no hope)	355	4.5
Restless or jumpy	301	4.0
Feel everything was an effort (have no energy)	309	4.1
Sad	342	4.5
**Total score**	538	7.2

#### Internal consistency

Cronbach’s alpha indicated excellent internal consistency (α = 0.89). Average inter-item covariance was 0.83.

#### Construct validity

The PCA demonstrated a unidimensional construct (Table 6). One component structure is apparent, as all variables load onto Component 1, and all values are positive. 70.1% of the variance in the model is explained by one component.

**Table 6:** Principal component analysis

Variable	Component 1
Nervous	0.432
Hopeless (have no hope)	0.463
Restless or jumpy	0.453
Everything was an effort (have no energy)	0.436
Sad	0.452

#### Convergent validity

**Fig. 1:**
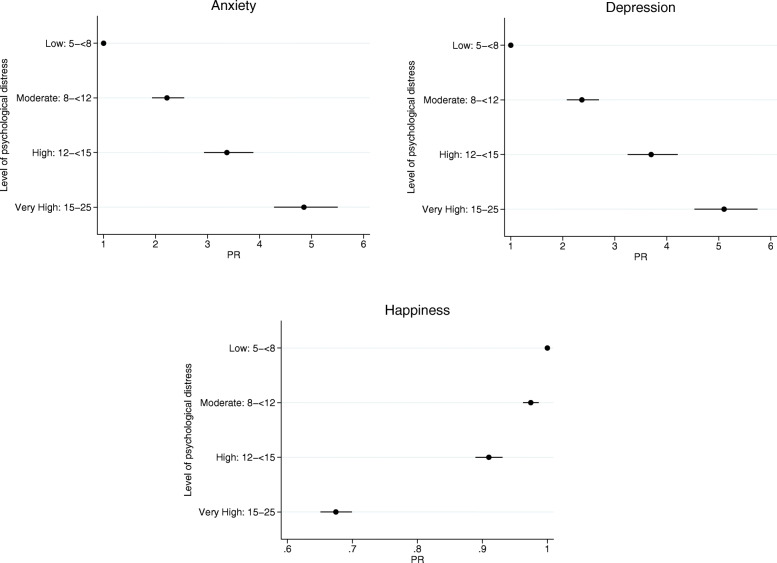
PR (95%CI) of depression, anxiety, and happiness by level of psychological distress

The MK-K5 achieved convergent validity with ever being diagnosed with depression, ever being diagnosed with anxiety, and happiness (Table 7, Fig. 1).

**Table 7:** Association between the MK-K5 and ever being diagnosed with depression, ever being diagnosed with anxiety, and high happiness (convergent validity assessment)

Level of psychological distress	Without outcome % (n)	With outcome % (n)	PR of reporting outcome (95%CI)
**Outcome: Ever diagnosed with depression**			
**MK-K5**			
Low (5- < 8)	87.3 (1801)	12.7 (262)	1 (ref)
Moderate (8- < 12)	69.9 (1535)	30.1 (660)	2.37 (2.08–2.70)
High (12- < 15)	53.0 (572)	47.0 (507)	3.70 (3.25–4.21)
Very High (15–25)	35.2 (581)	64.8 (1070)	5.10 (4.53–5.75)
**Outcome: Ever diagnosed with anxiety**			
**MK-K5**			
Low (5- < 8)	88.3 (1822)	11.7 (241)	1 (ref)
Moderate (8- < 12)	74.1 (1626)	25.9 (569)	2.22 (1.93–2.55)
High (12- < 15)	60.6 (654)	39.4 (425)	3.37 (2.93–3.88)
Very High (15–25)	43.3 (715)	56.7 (936)	4.85 (4.28–5.50)
**Outcome: High happiness**			
**MK-K5**			
Low (5- < 8)	97.2 (2001)	2.8 (57)	1 (ref)
Moderate (8- < 12)	94.8 (2075)	5.3 (115)	0.97 (0.96–0.99)
High (12- < 15)	88.5 (952)	11.5 (124)	0.91 (0.89–0.93)
Very High (15–25)	65.6 (1073)	34.4 (563)	0.67 (0.65–0.70)

Thirty-six percent of participants self-reported ever being diagnosed with depression, and 30% of participants self-reported ever being diagnosed with anxiety. The prevalence of depression was significantly higher among those with moderate, high and very high levels of psychological distress compared to those with low psychological distress (PR = 2.37, 95%CI = 2.08–2.70 for moderate psychological distress; PR = 3.70, 95%CI = 3.25–4.21 for high psychological distress; PR = 5.10, 95%CI = 4.53–5.75 for very high psychological distress).

The prevalence of anxiety was significantly higher among those with moderate, high and very high levels of psychological distress compared to those with low psychological distress (PR = 2.22, 95%CI = 1.93–2.55 for moderate psychological distress; PR = 3.37, 95%CI = 2.93–3.88 for high psychological distress; PR = 4.85, 95%CI = 4.28–5.50 for very high psychological distress).

Eighty-eight percent of participants reported high levels of happiness. It was less common for participants with moderate, high, and very high levels of psychological distress to report higher levels of happiness compared to those with low psychological distress (PR = 0.97, 95%CI = 0.96–0.99 for moderate psychological distress; PR = 0.91, 95%CI = 0.89–0.93 for high psychological distress; PR = 0.67, 95%CI = 0.65–0.70 for very high psychological distress) (Table 7, Fig. 1).

Across all three outcomes, findings were consistent with a dose-response relationship, with increasing psychological distress level associated with increasing prevalence of anxiety and depression, and decreasing prevalence of high happiness.

#### Divergent validity

**Fig. 2:**
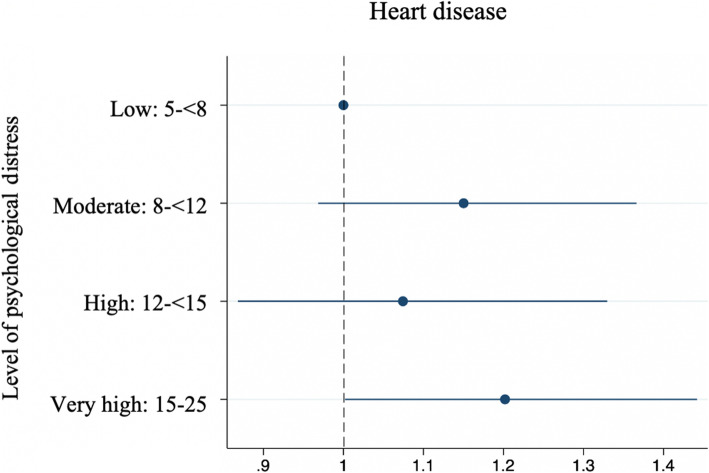
PR (95%CI) of heart disease by level of psychological distress

Eleven percent of Mayi Kuwayu Study participants reported a lifetime diagnosis of heart disease. The prevalence of self-reported heart disease was not significantly different among participants with moderate (PR = 1.15; 95%CI = 0.97–1.34) or high psychological distress (PR = 1.07; 95%CI = 0.87–1.33) compared to low psychological distress. The prevalence of heart disease was significantly higher among those with very high compared to low levels of psychological distress (PR = 1.20; 95%CI = 1.00–1.44) (Table 8, Fig. 2).

**Table 8:** Association between the MK-K5 and ever being diagnosed with Heart Disease (divergent validity assessment)

Level of psychological distress	No diagnosis of heart disease % (n)	Diagnosis of heart disease % (n)	PR of heart disease (95% CI)
Low (5- < 8)	89.8 (1853)	10.2 (210)	1 (ref)
Moderate (8- < 12)	88.3 (1938)	11.7 (257)	1.15 (0.97–1.34)
High (12- < 15)	89.1 (961)	10.9 (118)	1.07 (0.87–1.33)
Very High (15–25)	87.8 (1449)	12.2 (202)	1.20 (1.00–1.44)

#### Clinical utility

**Fig. 3:**
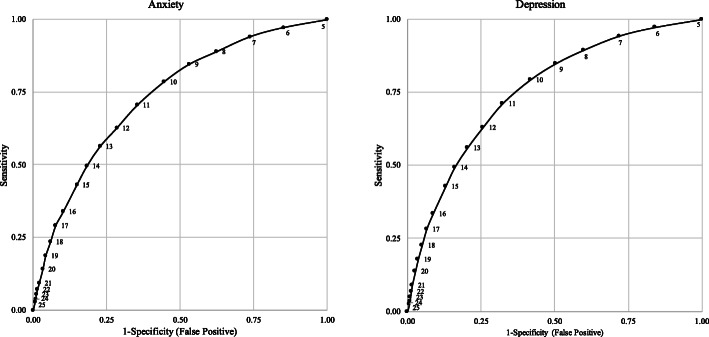
Result of ROC Curve Analyses: ever being diagnosed with depression and ever being diagnosed with anxiety

The MK-K5 cut-off score of 11/25 was identified as the optimum score in identifying self-reported cases of both depression and anxiety in this cohort (Fig. 3; Table 9).

**Table 9:** Measures of agreement for MK-K5 cut-off scores for ever being diagnosed with depression and ever being diagnosed with anxiety

	Sensitivity	Specificity	Positive Predictive Value	Negative Predictive Value
**MK-K5 Score**	**Diagnosis of Depression**			
≥ 7	94.36%	28.02%	13%	2%
≥ 8	89.52%	40.12%	15%	3%
≥ 9	84.95%	49.52%	17%	3%
≥ 10	79.31%	58.30%	19%	4%
**≥ 11**	**71.31%**	**67.61%**	**22%**	**4%**
≥ 12	63.11%	74.31%	25%	5%
≥ 13	56.14%	79.57%	27%	6%
≥ 14	49.46%	83.92%	31%	6%
**MK-K5 Score**	**Diagnosis of Anxiety**			
≥ 7	94.10%	26.39%	13%	2%
≥ 8	88.90%	37.82%	14%	3%
≥ 9	84.62%	47.02%	16%	3%
≥ 10	78.67%	55.45%	18%	4%
**≥ 11**	**70.57%**	**64.63%**	**20%**	**5%**
≥ 12	62.69%	71.58%	22%	5%
≥ 13	56.38%	77.25%	25%	6%
≥ 14	49.65%	81.73%	27%	6%

## Discussion

This is the first time that a comprehensive psychometric validation has occurred for the K5 in the Aboriginal and Torres Strait Islander population. The MK-K5, which includes minor wording changes and clarifying statements from the K5, is an acceptable, reliable, and valid measure of psychological distress with good clinical utility for Aboriginal and Torres Strait Islander adults in Australia. Assessment of face validity of the MK-K5 showed that participants understand the items in the MK-K5 and see them as a progression of intensity along one dimension. The MK-K5 demonstrated good acceptability, good internal consistency/reliability, no indication of item redundancy, and high homogeneity of items. Construct validity was achieved, with one underlying component that captured 70.1% of total variance. The MK-K5 showed evidence of convergent and divergent validity. MK-K5 cut-off scores of 11 or above indicate referral for further clinical assessment for risk of depression and/or anxiety. Future studies may investigate the finding that some MK-K5 concepts were interpreted positively by conducting focus groups. If such focus group testing reproduces the finding that some MK-K5 concepts are interpreted positively, then further modification to the MK-K5 may be necessary to ensure the measure is only looking at the negative aspects of the concepts.

Each item of the MK-K5 discussed in the focus groups achieved face validity for psychological distress. This supports the process that was used to tailor the measure in the Mayi Kuwayu Study. This also aligns with the original Aboriginal and Torres Strait Islander workshop that modified the K10 to become the K5 [16]. In the workshop, the K10 was culturally adapted to enhance understanding in the Aboriginal and Torres Strait Islander context. The adaption of the K5 to become the MK-K5 was based on the same reasons: to enhance the conceptual understanding of the K5 for Aboriginal and Torres Strait Islander people. This adaptation to become the MK-K5 was necessary because the initial K5 development only occurred over one workshop and was not comprehensive. The MK-K5 was further adapted and validated over a total of 30 focus groups across the country to represent the diversity of experiences and cultures of Aboriginal and Torres Strait Islander peoples.

Further, the face validity assessment in both the initial Mayi Kuwayu Study focus groups and those conduced in this study demonstrate that a purely statistical validation of a measure does not result in a culturally appropriate measure. The initial face validity assessment resulted in slight wording changes to the items in the K5 to increase Aboriginal and Torres Strait Islander participant understanding. The face validity assessment in this study endorsed these changes. This demonstrates the need for cultural validation, which can occur through focus group assessment, alongside the statistical validation of a measure.

It is important that measures are designed or tailored for Aboriginal and Torres Strait Islander people. Measures designed for the general Australian population are based in Western conceptions of health which often make them “culturally and socially inappropriate” for Aboriginal and Torres Strait Islander peoples [16]. Aboriginal and Torres Strait Islander people must be involved in the design, implementation and delivery of health services and strategies, including in the design of health measures, for the measures to be relevant [39]. Our findings indicate that participants found the MK-K5 acceptable: the vast majority of participants completed the MK-K5 items (< 5% missing individual items and < 10% missing total score).

Good internal consistency of the MK-K5 was achieved in the Aboriginal and Torres Strait Islander population (α = 0.89). This is likely due to the short nature of the MK-K5. Reducing the number of items in a measure increases the likelihood that it is only assessing one construct. Items from the longer K10 that were weakly correlated (or redundant) may now be absent in the K5 [34]. The result is consistent with previous literature on the reliability of the unmodified K5 [21].

The MK-K5 showed good construct validity in the Aboriginal and Torres Strait Islander population as determined through PCA. Uni-dimensionality of the MK-K5 is supported through focus group data. Participants revealed that the items used in the MK-K5 are linked sequentially, with some concepts occurring first before emotions and feelings are heightened, building into the other items. This indicates that the MK-K5 taps into one underlying construct, but the MK-K5 concepts relate to different stages of this construct.

The MK-K5 showed evidence of convergent validity with outcomes hypothesised to be linked, complementing previous literature on the convergent validity of the K6 and K10 with diagnoses of anxiety and affective disorders in other populations [40, 41]. Our findings also provided evidence of divergent validity, with a weak association observed between MK-K5 and heart disease. A weak association is expected because of the widespread impacts of psychological distress on all aspects of an individual’s health, wellbeing and other lifestyle outcomes.

The clinical utility of the MK-K5 in detecting a diagnosis of depression or anxiety was assessed, with the optimum cut-off score for the MK-K5 being 11/25. The Australian healthcare sector, and especially the mental health sector, is underfunded, under-resourced [38], and strained, with 12.4% of GP visits related to psychological concerns including depression and anxiety [42]. An MK-K5 cut-off score of 11/25 would maximise the entry of people who require referral for clinical psychological assessment into the system, while allowing true negative cases to be filtered out of the healthcare system or referred to other low intensity supports or interventions.

Our findings indicate that the MK-K5 score to appropriately identify Aboriginal and Torres Strait Islander people who require referral for clinical psychological assessment and treatment is 11. Applying the previous K5 categories to this sample, 39.1% (*n* = 2730) of participants are classified as having high/very high levels of psychological distress. However, applying a referral threshold score of 11 increases the proportion of participants classified as having high/very high levels of psychological distress to 46.3% (*n* = 3236). We acknowledge that participants with scores below 11 may also have depression or anxiety, and that not all participants with scores ≥11 have depression or anxiety. However, we recommend using the MK-K5 measure with a score of ≥11 for clinical referral for further investigation of depression and anxiety for Aboriginal and Torres Strait Islander peoples. This balances those entering into the healthcare system to receive the care they require, without overburdening an already strained system.

### Limitations

The Mayi Kuwayu Study is a national longitudinal study which was not designed to be representative; rather it was designed to capture a diversity of experiences. However, this will not impact on the robustness of reliability and validity testing. This sample is skewed to older Aboriginal and Torres Strait Islander peoples; however face validity was tested across all age groups. The focus groups were coded by a sole coder. While this may place some issues in the major themes extracted from the focus groups, these themes were discussed with colleagues/supervisors.

The study relied on self-report data, which may affect responses. Participants engaging with self-report data may respond to questions based on item properties in the measure or based on the social desirability to respond positively [43]. The Mayi Kuwayu Study was designed to mitigate these issues. First, the Mayi Kuwayu Study was developed with extensive community consultation to ensure it maintained cultural sensitivity and was accepted by Aboriginal and Torres Strait Islander communities. Secondly, the Mayi Kuwayu Study is designed to protect confidentiality and anonymity [23].

ROC curve analysis calls for gold standard data (i.e. a current diagnosis from a healthcare professional) for comparison with the MK-K5 [37]. There is no gold standard data available on clinically diagnosed anxiety or affective disorders in the dataset under study. Therefore, this analysis used self-reported lifetime diagnosis of anxiety or depression in place of a gold standard. However, due to the chronic nature of depression and anxiety [44, 45] the results still serve their purpose.

## Conclusion

Aboriginal and Torres Strait Islander people experience high levels of psychological distress due to historic and contemporary colonisation, trauma, racism, and marginalisation from society. The Kessler Psychological Distress Scales are used as a short-form screening measure of psychological distress in the Aboriginal and Torres Strait Islander population. This is the first national comprehensive validation study of a short-form Kessler Psychological Distress Scale in the Aboriginal and Torres Strait Islander population, with slight modifications to support participant comprehension. This study found that the MK-K5 has good face validity, is acceptable, has good internal reliability and validity, and is appropriate for use as a population level screening tool for psychological distress. It additionally has clinical utility for referral to assess anxiety and depression. Using the MK-K5, with a cut-off score of 11/25 to identify risk of anxiety and depression, has potential to more accurately identify Aboriginal and Torres Strait Islander people who need clinical support (when implemented and delivered in culturally appropriate ways), strengthening health and wellbeing assessment and referral.

## Supplementary Information

**Additional file 1: Table S1.** Questions from the Mayi Kuwayu Study. **Table S2.** Summary of Mayi Kuwayu Study focus groups.

## Data Availability

The datasets analysed during the current study are available via application from the Mayi Kuwayu Study Data Governance Committee, https://mkstudy.com.au/wp-content/uploads/2020/07/Data-Application-form.pdf

## Abbreviations

NATSIHS: National Aboriginal and Torres Strait Islander Health Survey
Mayi Kuwayu Study: Mayi Kuwayu the National Study of Aboriginal and Torres Strait Islander Wellbeing
K10: 10 items Kessler Psychological Distress Scale
K6: 6 item Kessler Psychological Distress Scale
K5: 5 item Kessler Psychological Distress Scale
MK-K5: Mayi Kuwayu Study modified 5 item Kessler Psychological Distress Scale
