# Supplementary material for: Reliability, validity, and clinical utility of a culturally modified Kessler scale (MK-K5) in the Aboriginal and Torres Strait Islander population
Source: BMC Public Health. 2021 Jun 10;21:1111. doi: 10.1186/s12889-021-11138-4 (PMC8194217; doi:10.1186/s12889-021-11138-4)
Supplement: Supplementary file 1 — Additional file 1: Table S1. Questions from the Mayi Kuwayu Study. Table S2. Summary of Mayi Kuwayu Study focus groups. [file 12889_2021_11138_MOESM1_ESM.docx]

**Supplementary material**

Table S1

*Questions from the Mayi Kuwayu Study*

| Name | Question | Response Options (score) | Categorisation |
| --- | --- | --- | --- |
| K5 | In the last 4 weeks about how often did you…  … feel nervous?  … feel hopeless (have no hope)?  … feel restless or jumpy?  … feel everything was an effort (have no energy)?  … feel sad? | All of the time (5)  Most of the time (4)  Some of the time (3)  A little of the time (2)  None of the time (1) | Low (score 5–<8)  Moderate (score 8–<12)  High (score 12–<15)  Very high (score 15-25) |
| Depression | Has a doctor ever told you that you have… | Depression | No  Yes |
| Anxiety | Has a doctor ever told you that you have… | Anxiety | No  Yes |
| Happiness | In the last 4 weeks about how often did you feel happy? | All of the time (5)  Most of the time (4)  Some of the time (3)  A little of the time (2)  None of the time (1) | Low happiness (none of the time, a little of the time)  High happiness (some, most, or all of the time) |
| Heart Disease | Has a doctor ever told you that you have… | Heart disease | No  Yes |

Table S2

*Summary of Mayi Kuwayu Study focus groups*

| **Focus Group** | **State/Territory** | **Location**  **(Urban, Regional, Remote)** | **Type of Organisation** | **Number of Participants** |
| --- | --- | --- | --- | --- |
| 1 | NSW | Regional |  | 25 |
| 2 |  |  |  |  |
| 3 |  |  |  |  |
| 4 |  |  |  |  |
| 5 | NSW | Regional |  | 9 |
| 6 | NT | Remote |  | 7 |
| 7 | NSW | Regional |  | 9 |
| 8 | ACT | N/A | National Conference | 25 |
| 9 |  |  |  |  |
| 10 |  |  |  |  |
| 11 | NSW | Regional |  | 7 |
| 12 | WA | Regional |  | 8 |
| 13 |  |  |  | 13 |
| 14 | QLD | Regional |  | 4 |
| 15 | QLD | Remote |  | 5 |
| 16 | NT | Regional |  | 4 |
| 17 | SA | Remote |  | 7 |
| 18 | ACT | Urban |  | 5 |
| 19 | WA | Regional |  | 10 |
| 20 | WA | N/A | National Conference | 6 |
| 21 |  |  |  | 9 |
| 22 |  |  |  | 5 |
| 23 |  |  |  | 7 |
| 24 |  |  |  | 8 |
| 25 |  |  |  | 4 |
| 26 |  |  |  | 9 |
| 27 | TAS | Urban |  | 8 |
| 28 | NT | Remote |  | 3 |
| **Total Number of Participants** | | | | 197 |
